# The Role of Animacy and Structural Information in Relative Clause Attachment: Evidence From Chinese

**DOI:** 10.3389/fpsyg.2019.01576

**Published:** 2019-07-17

**Authors:** Nayoung Kwon, Deborah Ong, Hongyue Chen, Aili Zhang

**Affiliations:** ^1^Department of English Language & Literature, Konkuk University, Seoul, South Korea; ^2^School of Foreign Languages, Shandong Jianzhu University, Jinan, China

**Keywords:** animacy, Chinese, relative clause, attachment ambiguity, SR/OR asymmetry

## Abstract

We report one production and one comprehension experiment investigating the effect of animacy in relative clause attachment in Chinese. Experiment 1 involved a fill-in-the-blank task that manipulated the order of an animate noun phrase in a complex NP construction. The results showed that while low attachment responses exceeded high attachment responses overall (cf. Shen, 2006), a tendency exists to attach a relative clause to an animate NP in Chinese (cf. Desmet et al., 2002). Experiment 2 used a rating task to examine the interplay between animacy and structural information by manipulating the order of the animate NP as well as the relative clause type (i.e., subject vs. object relative clauses). The results showed that the animate NP modification tendency found in Experiment 1 was limited to subject-relative clauses and that no animacy-related effect was found with object-relative clauses. These results are incompatible with purely structural parsing strategies such as Late Closure (Frazier, 1987) and the Predicate Proximity Principle (Gibson et al., 1996). Instead, the current results suggest that attachment ambiguity resolution in Chinese relative clauses is sensitive to animacy as well as structural information.

## Introduction

Previous studies have shown that languages have different relative clause attachment preferences (Cuetos and Mitchell, 1988) but that these preferences are modulated by various factors such as animacy (Desmet et al., 2002), prosody (Fodor, 1998, 2002), and language-internal grammatical factors (Hemforth et al., 2015). However, little is known whether and how these factors interact with each other. In this paper, we aim to investigate the interplay between animacy and structural information based on production and comprehension experiments of relative clause attachment preference in Mandarin Chinese.

**Figure 1:**
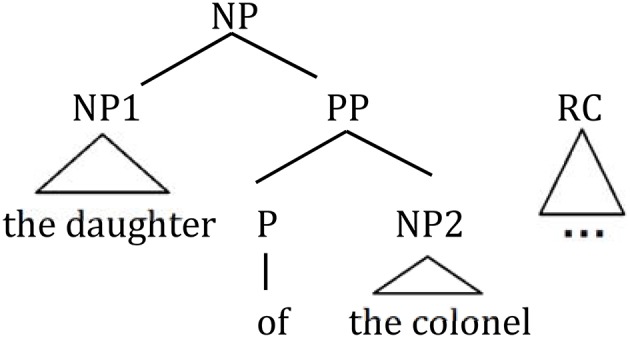
Phrase structure of sentence (1).

A relative clause is ambiguous when a complex NP occurs in its head noun position as in (1), as it can be interpreted to modify either NP1 (*the daughter;* high attachment, HA henceforth) or NP2 (*the colonel;* low attachment, LA henceforth) (see Figure 1).

(1) A sample experimental sentence from Cuetos and Mitchell (1988).

The journalist interviewed [_NP_ the daughter of the colonel [_RC_ who had had the accident]].

While Late Closure (Frazier, 1987) predicts that languages uniformly show an LA parsing bias, attaching a relative clause to a recently processed NP2, many studies have shown cross-linguistic variations in relative clause attachments. For example, based on experiments in Spanish and English, Cuetos and Mitchell (1988) showed that native Spanish speakers have an HA preference (see also Carreiras and Clifton, 1993; Gilboy et al., 1995; Dussias, 2001), interpreting a relative clause to modify NP1 while native English speakers have an LA preference (see also Carreiras and Clifton, 1993; Corley, 1995; Gilboy et al., 1995; Fernández, 2000; Dussias, 2001; Felser et al., 2007). Subsequent studies have provided further evidence for cross-linguistic differences by showing that Chinese (Shen, 2006), Arabic (Quinn et al., 2000), Basque (Gutiérrez-Ziardegi et al., 2004), Brazilian Portuguese (Miyamoto, 1998), Norwegian, Romanian, and Swedish (Ehrlich et al., 1999) have an LA preference, but that Korean (Jun, 2003; Jun and Kim, 2004; Lee and Kweon, 2004), Japanese (Kamide and Mitchell, 1997; Sturt et al., 1999; Miyamoto et al., 2004), Hindi (Vasishth et al., 2004), French (Zagar et al., 1997; Pynte, 1998; Frenck-Mestre and Pynte, 2000), German (Hemforth et al., 2000a,b; Konieczny and Hemforth, 2000), Dutch (Brysbaert and Mitchell, 1996), and Greek (Papadopoulou and Clahsen, 2003) have an HA preference.

Given these cross-linguistic differences, Cuetos and Mitchell (1988) argued that Late Closure is not a language-universal parsing strategy because different languages involve arbitrarily different parsing strategies. In response, Gilboy et al. (1995) argued that while structural parsing principles such as Late Closure are language-universal, parsing strategies are construction-specific; thus, language-universal strategies are irrelevant to non-primary phrases like relative clauses. In their proposal, structural parsing strategies like the Late Closure principle are relevant only to primary phrases, which include the main subject-predicate relation of a clause and its obligatory constituents. In contrast, they further argued that analyses of relative clauses are susceptible to non-structural as well as structural information. On the other hand, Gibson et al. (1996) argued that Late Closure is indeed a language-universal parsing principle relevant to relative clause attachment, but its effect is modulated by another factor: Predicate proximity. According to the Predicate Proximity Principle, a relative clause is preferably attached as closely as possible to a predicate, which would lead to an HA. Thus, in their proposal, cross-linguistic variations in relative clause attachments are based on parametric variations in the strength of the Predicate Proximity Principle relative to that of the Late Closure Principle in a given language; if the Predicate Proximity Principle is stronger than Late Closure, an HA preference is observed (as in Spanish), but if it is weaker, an LA preference is observed (as in English).

More recent studies have focused on factors more external to parsing principles such as language-internal differences, prosody, frequency and animacy. For example, Grillo and Costa (2014) argued that pseudo-relatives have different attachment biases from genuine relative clauses, and the apparent cross-linguistic differences in relative clause attachment biases are based on the availability of pseudo-relatives in a given language. Hemforth et al. (2015) also based their arguments on cross-linguistic grammatical differences, arguing that relative clause attachment bias in a given language is affected by focus and topic properties associated with the object and subject position in the language. On the other hand, Fodor (1998, 2002) proposed the implicit prosody hypothesis, which emphasizes the effect of prosody. The implicit prosody hypothesis claims that in silent reading, which occurs in most comprehension experiments conducted in the laboratory, default prosody affects relative clause attachment. However, pointing out that the default prosodic phrasing in English predicts an HA preference contrary to the LA preference actually observed in English, Jun (2010) argued that the effect of prosody is modulated by the length of a relative clause and grammatical properties of a relative clause verb only when the relative clause is short (see also Yao and Scheepers, 2018 and references therein).

More relevant to the current study is Desmet et al. (2002; cf. Gilboy et al., 1995), which investigated the effect of animacy. Previously, Mitchell and Brysbaert (1998) found that corpus frequencies of Dutch relative clauses did not correlate with the reading time results reported in Brysbaert and Mitchell (1996). These results are problematic for experience-based accounts such as the Tuning Hypothesis, which argues that initial structural decisions are based on the relative frequency with which a language user encounters a certain structure (Mitchell and Cuetos, 1991; Mitchell et al., 1995). However, noting that the relative clauses examined in Mitchell and Brysbaert (1998) mostly involved a non-human NP1 while most of the experimental stimuli in Brysbaert and Mitchell (1996) involved a human NP1, Desmet et al. argued that the apparent discrepancy of reading times and frequencies was due to the animacy effect. Based on carefully conducted corpus analyses, they showed that in Dutch, a non-human NP1 is frequently associated with an LA bias, while a human NP1 is frequently associated with an HA bias, which accounted for the contrasting results in Brysbaert and Mitchell (1996) and Mitchell and Brysbaert (1998). In addition, Desmet et al. showed that the patterns of the corpus frequencies corresponded to the attachment preferences found in their production experiments (see also Desmet et al., 2006 for compatible results in comprehension experiments).

However, while the effect of animacy in Dutch is convincing, to our knowledge, no study to date has examined the interplay between animacy and the grammatical role of the head NP within a relative clause. Hemforth et al. (2015) examined the effect of the grammatical role of the head NP within the main clause but did not consider grammatical roles within the relative clause or the effect of animacy. Desmet et al. examined the effect of animacy but not the effect of structural factors. However, previous studies have shown that the animacy properties of a head NP are closely related to its relative clause type (i.e., the grammatical role of a head NP within a relative clause). For example, in German and Dutch, subject-extracted relative clauses (SRs) typically involve an animate head NP, while object-extracted relative clauses (ORs) typically involve an inanimate head NP (Mak et al., 2002). Similar patterns of results were found in Chinese as well. In a corpus study of the Chinese Treebank Corpus 5.0 (Palmer et al., 2005), Wu et al. (2010) showed that 86.7% of the object-extracted relative clauses examined occurred with an inanimate head NP while 64.5% of the subject-extracted relative clauses examined occurred with an animate head NP (see also Pu, 2007 for similar results). While more fine-grained details differ for Pu (2007) and Wu et al. (2010), these studies suggest that an animate NP is more likely to occur as the head NP of a subject relative clause but is dispreferred as the head NP of an object relative clause (see also Lin and Hu, in press).

In the present paper, we address two specific questions. First, does animacy affect relative clause attachment? Second, if so, does animacy interact with the grammatical role of a potential head NP within a relative clause? The first question is investigated in Experiment 1 with a fill-in-the-blank production task. Then, we investigate the second question in Experiment 2 with a comprehension task. Both experiments employ structurally ambiguous relative clause sentences in Mandarin Chinese.

## Experiment 1: Production

**Figure 2:**
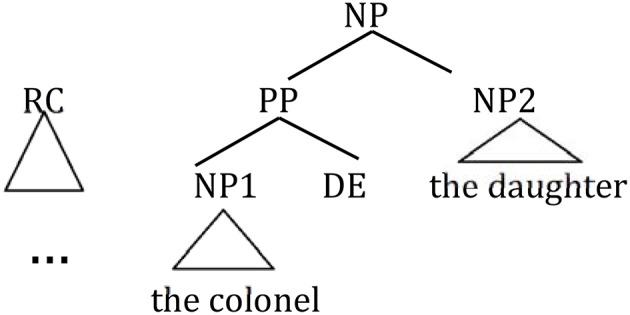
Phrase structure of sentence (1) in Chinese presented in English.

The goal of Experiment 1 was to examine the effect of animacy in relative clause attachment in Chinese. A relative clause in Chinese may be ambiguous just like in English or Spanish; when a complex NP (e.g., *NP1 de NP2*, “NP2 of NP1”) occurs in its head noun position, the relative clause can be interpreted as modifying either NP1 or NP2. However, in Chinese, modification of NP1 results in low attachment, and modification of NP2 results in high attachment due to differences in branching directions from those in English in this particular construction (see Figure 2). To avoid any confusion due to this difference, attachments are described in terms of HA or LA instead of (or in conjunction with) NP1 or NP2 attachment throughout this paper.

Experiment 1 created two experimental conditions by manipulating the animacy of a potential head NP in a complex NP (NP1-de NP2, “NP2 of NP1”). In the Animate-Inanimate condition, NP1 was animate while NP2 was inanimate. The Inanimate-Animate condition reversed the order of the NPs, as shown in (2). The target construction was preceded by a blank line and *de*, signaling participants to fill in the blank with a prenominal relative clause modifying the following complex NP.

(2) Target items of Experiment 1
  a. Animate-Inanimate condition
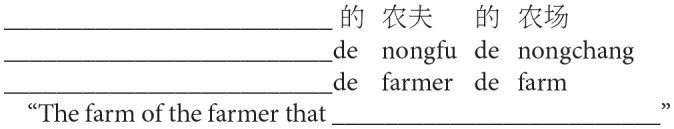
  b. Inanimate-Animate condition:
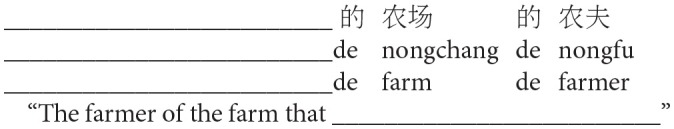

If animacy affects relative clause attachment in Chinese, we predict an LA preference (i.e., NP1 attachment) in the Animate-Inanimate condition and an HA preference (i.e., NP2 attachment) in the Inanimate-Animate condition. On the other hand, if animacy does not affect relative clause attachment in Chinese, the LA bias previously found in Chinese (Shen, 2006) predicts a consistent LA bias regardless of the animacy ordering. Alternately, the animacy effect might interact with the LA preference in Chinese. Shen (2006) did not manipulate animacy, employing human NPs in most of the experimental sentences. Thus, straightforward predictions cannot be made based on that study. However, if these two factors interact, then we predict a stronger LA tendency for the Animate-Inanimate condition than for the Inanimate-Animate condition. For the latter condition, the LA bias should be reduced or even absent if the animacy effect cancels it in Chinese.

### Methods

#### Participants

Forty native speakers of Mandarin (mean age = 20.3, SD = 2.5) participated in the production study. All of participants were born and raised in China, although 20 of the participants went to Singapore at an average age of 21.2 years (SD = 3.3). At the time of the experiment, all the participants were all registered at a university either in Singapore or in China as an undergraduate or graduate student.

#### Materials

Twenty four sets of target items like (2) were constructed. Two questionnaires were created using a Latin square design, each with 12 target items per condition. There were also 26 filler items with one NP preceded by *de* and a blank line. Among the 26 filler items, 13 included an animate NP and the other 13 an inanimate NP. The two lists were pseudo-randomized such that no two items with the same condition would be adjacent to each other.

#### Procedures

The production study was a paper-and-pencil task asking participants to complete the phrases as they desired by filling in the blank. There was no time limit, but most participants took <30 min.

#### Data Analysis

There were technical errors in the presentation of two items, so they were excluded from the analyses. Four raters independently judged the remaining responses. As Chinese does not have morphological subject-verb agreement, decisions were based on plausibility (e.g., HA interpretation: ‘*the farmer of the farm who got married last year*’ or LA interpretation: ‘*the farmer of the farm that is fertile’*). When ambiguous (e.g., ‘*the farmer of the farm that I like*’), responses were coded as unclassified (UC). In case of disagreement, the raters re-examined the cases together to reach a consensus. For most cases with such disagreement, however, the responses were conservatively coded as UC.

Analyses were first run on classified (i.e., including both LA and HA) vs. unclassified responses using a generalized Linear Mixed Effect (LME) model with a binomial distribution (Baayen, 2008; Baayen et al., 2008; Jaeger, 2008). The lme4 R package (Bates et al., 2015; Version 1.1–8) was used. The regression included the experimental condition (NP ordering: Animate-Inanimate condition vs. Inanimate-Animate condition) as the within-subjects predictor. The fixed-effect factor was coded numerically using sum coding. The regression models incorporated crossed random intercepts for items and participants. Models were constructed with the maximal random effect structure and progressively simplified when the models did not converge (Barr et al., 2013). The analyses yielded coefficients, standard errors and z-values for each fixed effect and interaction. *P*-values were calculated from the *Z*-score. The results showed that the number of UC responses did not greatly vary across the experimental conditions (all *p* > 0.88; Table 1). Given this, all subsequent analyses were run on classified completions (HA or LA) based on similar procedures described above. The regression for attachment responses, however, also included the group (Chinese participants recruited from Singapore vs. China) as the between-subjects predictor to examine whether there were group differences that could be based on language use, education, or other factors, in addition to the experimental condition (NP ordering: Animate-Inanimate condition vs. Inanimate-Animate condition) as the within-subjects predictor. The “slope” column in Table 2 indicates whether a random slope parameter corresponding to a fixed-effect factor was included in the model.

**Table 1:** Target responses by participants in Experiment 1.

	**Unclassified**	**Low attachment**	**High attachment**
Ani-Inani	56	336	37
Inani-Ani	84	200	145

**Table 2:** Generalized Linear Mixed Effects results for all participants in Experiment 1.

	***Estimate***	***SE***	***z***	***p***	**Slope**
(Intercept)	1.673	0.35	4.648	0.001	
Condition	0.989	0.21	4.711	0.001	(*p*)
Group	0.015	0.449	0.034	0.973	
Condition*Group	0.541	0.301	1.79	0.1	

*Coefficients, standard errors, z-values and p-values are reported for the main effects of Condition and Group manipulation, as well as for the interaction of these two factors. The “Slope” column indicates whether the random slope parameter corresponding to the effect was included in the model for participants (p) or items (i)*.

### Results and Discussion

All the attachment responses are summarized in Table 1. Statistical analysis results are presented in Table 2.

The results showed a significant main effect of NP ordering (*p* < 0.001) but no group effect. These results suggest that the order of animate NP affected attachment preferences regardless of group. That is, while LA responses outnumbered HA responses overall, confirming the LA preference in Chinese (Shen, 2006), the Animate-Inanimate condition elicited more LA (NP1 attachment) responses and fewer HA (NP2 attachment) responses relative to the Inanimate-Animate condition. Accordingly, the LA-HA response difference was bigger in the Animate-Inanimate condition than in the Inanimate-Animate condition.

On the other hand, when we ran a binomial test (R Core Team, 2018) to test a hypothesis that the observed proportion of LA responses does not differ from chance (i.e., 50% LA responses and 50% HA responses), the results showed that both conditions elicited significantly more LA responses than predicted by chance (*p* < 0.005) (Animate-Inanimate condition: 90.1%, 336 out of 373 attachment responses; Inanimate-Animate condition: 57.9%, 200 out of 345 attachment responses). Overall, these results suggest that the LA preference in Chinese (Shen, 2006) is confirmed in both NP ordering conditions, but that such tendency is stronger in the Animate-Inanimate condition than in the Inanimate-Animate condition. Thus, the effect of the animate NP ordering suggests that animacy plays an important role in relative clause attachment in Chinese. In particular, the results suggest that animate NPs are more likely to be modified by a relative clause than inanimate NPs are. Here, we will call this effect the animacy effect.

In addition, given our interest in the potential interaction of animacy and relative clause type (c.f. Pu, 2007; Wu et al., 2010), we further examined modification types of the two experimental conditions. To this aim, we examined the Low and High attachment responses presented in Table 1, focusing on data from 20 participants.^1^ The results are presented in Table 3.

**Table 3:** Responses by modification type.

**Animate-Inanimate**						**Inanimate-Animate**					
**Low attachment**			**High attachment**			**Low attachment**			**High attachment**		
DR^*^	**SR**	**OR**	**DR**	**SR**	**OR**	**DR**	**SR**	**OR**	**DR**	**SR**	**OR**
137	23	3	18	2	0	85	13	2	43	16	0

* *DR, Descriptive relative clause*.

Almost all responses were descriptive relative clauses, which are based on a simple attributive adjectival phrase preceding *de* (Li and Thompson, 1989, p. 118)^2^. There were also 54 cases of subject relative clauses and five cases of object relative clauses. Overall, the majority of these relative clauses modified NP1 (low attachment) regardless of its animacy (descriptive relative clauses: 78.4%, 222 out of 283 responses; subject relative clauses: 66.7%, 36 out of 54 responses; object relative clauses: 100%, 5 out of 5 cases). Thus, these results seem to suggest that animacy does not interact with relative clause type in Chinese, contra Pu (2007) and Wu et al. (2010). However, the low attachment tendency appeared to be stronger for the Animate-Inanimate condition (descriptive relative clauses: 88.3%, 137 out of 155 responses; subject relative clauses: 92%, 23 out of 25 responses; cf. object relative clauses: 100%, 3 out of 3 cases) than for the Inanimate-Animate condition (descriptive relative clauses: 66.4%, 85 out of 128 responses; subject relative clauses: 44.8%, 13 out of 29 responses; cf. object relative clauses: 100%, 2 out of 2 cases). Likewise, there was a stronger tendency to modify NP2 (high attachment) in the Inanimate-Animate condition (descriptive relative clauses: 33.6%, 43 out of 128 responses; subject relative clauses: 55.2%, 16 out of 29 responses; cf. object relative clauses: 0%, 0 out of 2 cases) than in the Animate-Inanimate condition (descriptive relative clauses: 11.6%, 18 out of 155 responses; subject relative clauses: 0.08%, 2 out of 25 responses; cf. object relative clauses: 0%, 0 out of 3 cases). These results seem to suggest that the animacy effect in Chinese relative clause attachment interacts with relative clause type. Thus, in Experiment 2, we aimed to investigate the interaction of animacy and relative clause type using a comprehension task.

To summarize, the production results suggest that the animacy effect interacts with the LA preference in Chinese. That is, while the results confirm the previously proposed LA preference in Chinese (Shen, 2006), they also suggest a tendency to use a prenominal relative clause to modify an animate rather than inanimate NP. Thus, the animate NP modification tendency becomes stronger when the resulting relative clause attachment coincides with the LA preference in Chinese. On the other hand, when the animate NP modification tendency conflicts with the LA tendency, as in the Inanimate-Animate condition, the LA preference becomes weaker. Based on the results of Experiment 1, we investigate the interaction between animacy and structural factors in Experiment 2.

## Experiment 2: Comprehension

The results of Experiment 1 supported an animate NP modification tendency in Chinese. Experiment 2 investigates whether this animacy effect interacts with the grammatical role of a head NP within a relative clause. Although there were too few object relative clauses in Experiment 1 to test this argument, previous studies have suggested that in Chinese, object-extracted relative clauses are typically associated with an inanimate head NP, while subject-extracted relative clauses are typically associated with an animate head NP (Pu, 2007; cf. Wu et al., 2010 and Lin and Hu, in press for such tendency for subject-modifying subject-extracted relative clauses). This means that the grammatical function of a head NP within a relative clause closely correlates with its animacy. In addition, the relative frequencies of animate head NPs in subject and object relative clause sentences have been shown to affect the processing difficulty of these sentences during online language processing. That is, the relative processing disadvantage of object relative clauses compared to subject relative clauses is (partially) modulated by the animacy of head NPs (Wu et al., 2012; for discussion of relevant issues in relative clause processing, see also Lin and Bever, 2006; Kwon et al., 2010, 2013). Thus, the interplay between animacy and relative clause type may also affect ambiguity resolution of relative clause attachment. In particular, we predict that the animate NP modification tendency found in Experiment 1 would be restricted to subject-extracted relative clauses, and that for object-extracted relative clauses, no such pattern or even an inanimate NP modification tendency would be observed.

To investigate these questions, Experiment 2 used a rating method, varying the order of animate and inanimate NPs in a complex NP construction (NP1-de NP2, “NP2 of NP1”) as in Experiment 1: “animate NP1- inanimate NP2” vs. “inanimate NP1- animate NP2.” In addition, the complex NP was preceded by either a subject- (SR) or object-extracted (OR) relative clause. Thus, four experimental conditions were created in total, as shown in (3). In the experiment, the relative clauses and their head NPs always occurred as adverbials to avoid any potential effects of focus or topic properties associated with an argument position (see Hemforth et al., 2015).

(3) Target items of Experiment 2
  a. SR Animate-Inanimate condition
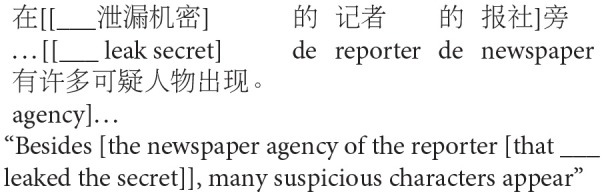
  b. SR Inanimate-Animate condition
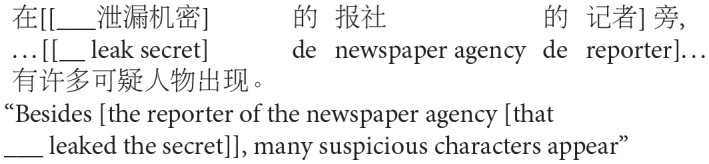
  c. OR Animate-Inanimate condition
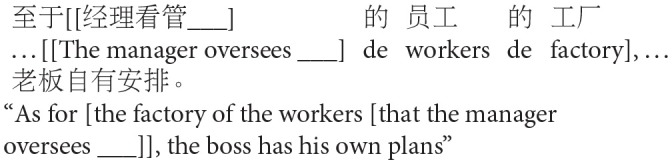
  d. OR Inanimate-Animate condition
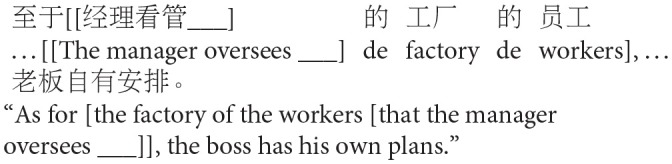

In accordance with the results of Experiment 1, we predict that animacy will affect relative clause attachment biases, modulating the LA tendency in Chinese. However, if the animacy effect interacts with the relative clause type, as SRs are typically associated with an animate head NP, the SR Animate-Inanimate condition will show a stronger LA preference (i.e., NP1 attachment) compared to the SR Inanimate-Animate condition, which might even show an HA preference (i.e., NP2 attachment). For ORs, the opposite patterns are predicted. As ORs are typically associated with an inanimate head NP, the OR Inanimate-Animate condition will show a stronger LA preference (i.e., NP1 attachment) compared to the OR Animate-Inanimate condition, which might even show an HA preference (i.e., NP2 attachment). On the other hand, if the animacy effect does not interact with relative clause type, then we predict that the animacy effect will be observed for both SR and OR conditions. Thus, the Animate-Inanimate condition will show a stronger LA preference (i.e., NP1 attachment) compared to the Inanimate-Animate condition regardless of whether the RC is an SR or an OR.

### Methods

#### Participants

Thirty two native Mandarin Chinese speakers in China (mean age = 20.01, *SD* = 1.01) participated in the comprehension study. At the time of the experiment, they were all registered at a university in China.

#### Materials

Twenty four sets of target items such as (3) were constructed (See Appendix in Supplementary Material). As experimental sentences differ across the conditions, we first conducted a norming test to control for plausibility. For example, for the sentences in (3) we created four norming test sentences as shown in (4), by placing a head NP in the gap position [i.e., the underlined position in (3)] within the relative clause.

(4) Items for plausibility test
  a. SR Animate condition
    记者泄漏了机密。
    “The reporter leaked the secret”
  b. SR Inanimate condition
    报社泄漏了机密。
    “The newspaper agency leaked the secret”
  c. OR Animate condition
    经理看管员工。
    “The manger oversees the workers.”
  d. OR Inanimate condition
    经理看管工厂。
    “The manager oversees the factory”

The four conditions of each item were distributed across four lists according to a Latin square design, along with 25 filler sentences. Sixteen native speakers of Chinese participated in the study (mean age = 21.3, *SD* = 0.68) and were asked to rate each sentence for its plausibility, with 1 if the sentence sounds very implausible and 5 if the sentence sounds very plausible. The results of the norming study appear in Table 4 below. The data were analyzed with Linear Mixed Effects, with RC type and NP ordering as within-subjects predictors. The initial model treated the OR Animate-Inanimate condition as the reference level for comparison with other conditions. The remaining analysis procedures were similar to those in Experiment 1. The results of the analyses are presented in Table 5. The results showed that all four conditions were all highly plausible and did not differ from each other in terms of plausibility.

**Table 4:** Plausibility mean ratings and standard error (in parentheses) in Experiment 2.

	**SR**		**OR**	
	**Animate**	**Inanimate**	**Animate**	**Inanimate**
Plausibility ratings	3.97 (0.125)	3.93 (0.121)	3.86 (0.11)	4.01 (0.114)
Acceptability ratings	2.56 (0.15)	2.81 (0.15)	2.54 (0.15)	2.56 (0.16)

**Table 5:** Linear mixed effect model results for norming study in Experiment 2.

	***Estimate***	***SE***	***t***
**PLAUSIBILITY**			
(Intercept)	3.943	0.2193	17.97
RC type	−0.005	0.0401	−0.13
NP order	−0.026	0.0401	−0.65
RC type *NP order	−0.047	0.0401	−1.17
**ACCEPTABILITY**			
(Intercept)	2.619	0.1979	13.23
RC type	−0.065	0.0659	−0.99
NP order	−0.068	0.0731	−0.93
RC type*NP order	0.043	0.0782	0.55

*Coefficients, standard errors (SE), and t-values are reported for the main effects of the RC type, the animate NP order, as well as the interaction of these two factors. The column for slope was removed, as no random slopes were justified*.

In addition, we conducted a norming test to control for acceptability of the target phrases. We created four norming test phrases, as shown in (5), by extracting the critical NP1 de NP2 phrase along with its modifying relative clause.

(5) Items for acceptability norming test
  (a) SR Animate-Inanimate condition
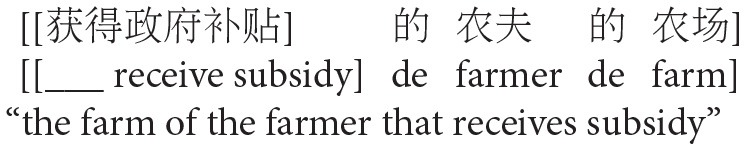
  (b) SR Inanimate- Animate condition
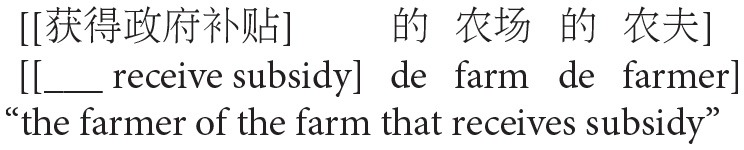
  (c) OR Animate-Inanimate condition
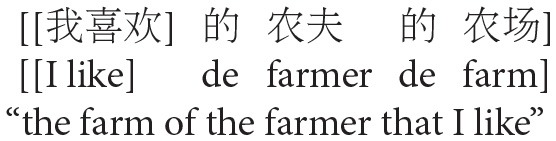
  (d) OR Inanimate-Animate condition
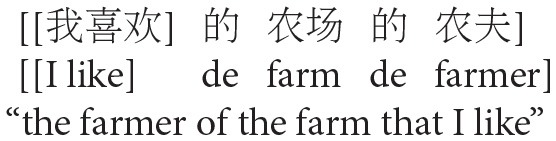

Four questionnaires were created using a Latin square design, each with 24 target items per list, along with 48 filler items of similar length. Twenty native speakers of Chinese (mean age = 20.5, *SD* = 0.69) rated each sentence on a scale of 1 to 5 for its acceptability, with 1 if the sentence sounded very strange and 5 if the sentence sounded very natural. One participant rated all the sentences (including simple filler items such as 戴着珍珠项链的女星 “an actress wearing a pearl necklace”) to be 1 or 2. As it was not clear whether he/she read sentences clearly, we removed the data from that participant. Data from the remaining 19 participants underwent statistical analysis procedures analogous to those used in the plausibility norming test. The results showed a marginal effect of an animacy ordering (β = −0.12426; *SE* = 0.06941; *t* = −1.8). Although the effect was not significant, the marginal effect suggests that the experimental sentences with a different NP ordering might differ in their acceptability. Thus, to avoid potential effects of acceptability, we removed the four items with the largest acceptability differences between the two NP orderings (mean difference of the four removed items = 2.7, *SD* = 0.35; mean difference of the remaining 20 items = 0.66, *SD* = 0.49). Statistical analyses of the acceptability ratings with the remaining 20 sets of items did not show any effect of acceptability. The results of the norming study and the statistical analyses are presented in Tables 4, 5, respectively.

Based on the results, we proceeded to Experiment 2. For 20 experimental sentences with 4 conditions, four lists were created using a Latin square design. There were 48 additional filler items of similar complexity and length. The four lists were pseudo-randomized such that no two items of the same condition would be adjacent to each other. On the questionnaire, each experimental item was followed by its LA and HA interpretations. For example, for sentence (3) a above, its HA interpretation (“The newspaper agency leaked the secret”) and its LA interpretation (“The reporter leaked the secret”) followed the sentence, along with a seven-point Likert scale. All sentences were presented in Chinese in the experiment, but a sample item is presented in its English translation in (6). The order of presentation of HA and LA interpretations was counterbalanced.

(6) A sample experimental item translated into English

“Given the sentence below, how likely are the following sentences to be true?”

Sentence: Besides the newspaper agency of the reporter that leaked the secret, many suspicious characters appear.


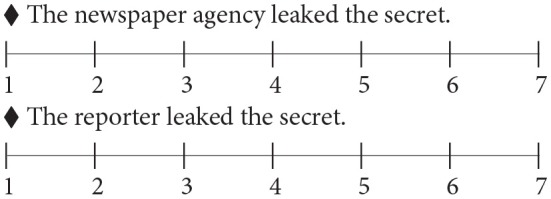


Filler sentences also involved relative clauses with *de*, but they did not have structural ambiguity. Questions for filler sentences were based on their interpretation. For example, for “The little soldier who was looked down on by the sergeant was blown away along with forest trees by the tornado,” participants were asked to rate the likelihood of “The sergeant looked down on the little solider” and “The sergeant looked down on the forest trees” being true.

#### Procedures

Participants were asked to read each item on the questionnaire and to rate how likely each interpretation was to be true given a sentence on a seven-point Likert scale. As in Experiment 1, there was no time limit, but most participants took <30 min.

#### Data Analysis

We removed data from 3 subjects, who rated all the sentences (including filler items) to be the same (either all 7 or 1), as it was not clear whether they had read the sentences clearly. With the remaining data, we first obtained rating differences between LA and HA interpretations by subtracting the rating of HA interpretations from LA interpretations. Accordingly, a positive difference suggests an LA preference, and a negative one suggests an HA preference. In addition, absolute values of rating differences would reveal the relative strength of the attachment preference: the higher the absolute value, the stronger the preference. We statistically analyzed these rating differences with Linear Mixed Effect Regression (LMER) analysis (Baayen, 2008; Baayen et al., 2008; Jaeger, 2008). The regression included the experimental conditions (RC type and animacy ordering) as within-subjects predictors and subjects and items as crossed random effects. The analysis yielded coefficients, standard errors, and *t*-values for each fixed effect and interaction. For the linear models, a given coefficient was judged to be significant at α = 0.05 if the value of |*t*| exceeded 2 (Baayen, 2008). The remaining analysis procedures were similar to those in Experiment 1.

### Results and Discussion

The overall results are given in Table 6, and statistical analysis results are presented in Table 7.^3^ There was neither a main effect of the NP ordering nor that of the RC type. However, there was a significant interaction between the two (*t* = −2.62), suggesting that the RC type modulated the effect of the animate NP ordering. Indeed, follow-up pairwise comparisons showed that the NP ordering effect was (marginally) significant for the SR conditions [*t*_1(1, 28)_ = 2.71, *p* < 0.012; *t*_2(1, 19)_ = 1.96, *p* < 0.066)] but not significant at all for the OR conditions [t_1(1, 28)_ = 0.36, n.s; *t*_2(1, 19)_ = 0.51, n.s.).

**Table 6:** Mean ratings and standard error (in parentheses) in Experiment 2.

	**SR**		**OR**	
	**Ani-inani**	**Inani-ani**	**Ani-inani**	**Inani-ani**
LA (NP1) ratings	6.19 (0.13)	5.47 (0.19)	5.81 (0.16)	5.77 (0.16)
HA (NP2) ratings	4.57 (0.21)	5.14 (0.18)	5.45 (0.17)	5.26 (0.17)
LA—HA difference	1.62 (0.29)	0.33 (0.32)	0.36 (0.27)	0.51 (0.28)

*A shaded cell indicates a significant difference between the Animate-Inanimate and the Inanimate-Animate condition*.

**Table 7:** Linear mixed effect model results for Experiment 1.

**LA vs. HA rating difference**	***Estimate***	***SE***	***t***	***slope***
(Intercept)	0.704	0.301	2.338	
RC type	−0.244	0.144	−1.695	(p)
NP Order	0.272	0.287	0.947	(p,i)
RC type*NP Order	−0.359	0.137	−2.619*	

*Coefficients, standard errors (SE), and t-values are reported for the main effects of the RC type, the animate NP order, as well as the interaction of these two factors. The “slope” column indicates whether the random slope parameter corresponding to the effect was included in the model for participants (p) or items (i). An asterisk indicates that the effect is significant at α = 0.05 (using the |t| > 2 criterion)*.

These results are compatible with the results in Experiment 1, where the animacy effect interacted with the default LA preference in Chinese. While all the conditions numerically showed an LA tendency (see Table 6), the LA preference was stronger when the animate NP modification tendency coincided with the default LA preference in Chinese, as in the case for the SR Animate-Inanimate condition. On the other hand, ORs did not show a clear animacy effect.

In summary, predictions were partially confirmed. An animate NP modification tendency interacted with relative clause type, but the NP ordering effect was significant only for SRs. On the other hand, there was no clear evidence that the animacy effect modulated relative clause attachments for ORs.

## General Discussion and Conclusion

The current study aimed to investigate a relative clause attachment preference in Mandarin Chinese by focusing on the interplay between animacy and structural information. To this aim, in Experiment 1, we first investigated whether animacy plays a role in relative clause attachment. The results showed more LA than HA responses elicited overall, confirming an LA preference in Chinese (Shen, 2006). However, a preference was also found to attach a relative clause to an animate NP rather than to an inanimate NP, suggesting that animacy affects relative clause attachment in Chinese. Based on the results of Experiment 1, Experiment 2 examined the interplay of animacy and structural information using a rating task. The results showed that the animacy effect found in Experiment 1 was constrained by RC type. That is, with SR sentences, the Animate-Inanimate condition elicited a significantly stronger LA preference than the SR Inanimate-Animate condition. On the other hand, for OR sentences, there was no such animacy effect. These results suggest that the effect of animacy is limited depending on the RC type. The implications of these findings are discussed below.

The results of the current study partially confirmed the animacy effect in relative clause attachment reported in Desmet et al. (2002). That is, similar to Dutch, relative clause attachment in Chinese is also affected by animacy of a potential head NP. However, it is not clear whether the animacy effect in Chinese is identical to that in Dutch. In Dutch, animacy (or humanness) of NP1 fully modulated attachment preferences such that the conditions with an animate NP1 (i.e., the animate NP1-animate NP2 condition and the animate NP1-inanimate NP2 condition) showed a NP1 attachment bias, while the conditions with an inanimate NP1 (i.e., the inanimate NP1-animate NP2 condition and the inanimate NP1-inanimate NP2 condition) showed a NP2 attachment bias (see study 1 in Desmet et al.). The current study of Chinese included only two of the four conditions examined in Dutch, and so direct comparison is not possible. Nonetheless, the effect of animacy in Chinese seems more limited than in Dutch, given that the inanimate NP1 condition did not show a clear NP2 attachment bias in Experiment 1. On the contrary, while fewer LA responses were elicited in the inanimate NP1 condition than in the animate NP1 condition, more LA responses than HA responses occurred in both conditions. However, the goal of this study was not to compare the animacy effect of Chinese to that of Dutch. Further research is required to understand exact differences in animacy effects in these two languages. Here, it suffices to say that the animacy of a potential head NP affects relative clause attachment in Chinese, as it does in Dutch.

Experiment 2 further showed that the animacy effect does not apply uniformly; rather, it is modulated by the relative clause type—specifically, the grammatical role that the head NP plays within a relative clause. This suggests that relative clause attachment in Chinese is constrained by the interplay of semantic (i.e., animacy) and structural (i.e., grammatical role) factors, among others. While the current study is limited in its scope and cannot account for parsing mechanisms underlying the LA bias in Chinese in general, the overall experimental results are not predicted by the Late Closure or the Predicate Proximity Principles, which predict a uniform attachment tendency across different types of relative clauses, regardless of the animacy of a potential head NP. In addition, our results may be incompatible with the Tuning hypothesis. As discussed briefly in the introduction, the Tuning Hypothesis predicts that initial structural decisions are based on language users' prior linguistic experiences (Mitchell and Cuetos, 1991; Mitchell et al., 1995). In the context of relative clause attachment, Desmet et al. (2002) performed a production study that showed a strong correspondence between corpus frequencies and relative clause attachment biases. This correspondence was confirmed in a subsequent comprehension study (Desmet et al., 2006). In Chinese, corpus studies have shown that SRs typically occur with an animate head NP, while ORs typically occur with an inanimate head NP (Pu, 2007; Wu et al., 2010 and Lin and Hu, in press for such tendency for subject-modifying subject-extracted relative clauses). Thus, the Tuning Hypothesis would incorrectly predict that in Experiment 2, animacy manipulation would fully interact with the RC type, such that the SR animate-inanimate condition and the OR inanimate-animate condition would favor an LA (i.e., NP1 attachment) while the SR inanimate-animate condition and the OR animate-inanimate condition would favor an HA (i.e., NP2 attachment). These predictions, however, were only partially supported, as the animacy effect was only significant in the comparison of the SR Animate-Inanimate condition and the SR Inanimate-Animate condition. On the other hand, for OR conditions there was no clear evidence that attachment preferences were modulated by animacy information. However, before we reject the Tuning Hypothesis based on these results, note that these apparent discrepancies may be due to “a grain problem” (see Mitchell et al., 1995 for a relevant discussion). For example, corpus results in Desmet et al. are based on all types of relative clause with a complex head NP. On the other hand, Wu et al.'s data are based on subject- and object-modifying SRs and ORs, and our experimental stimuli are based on SRs and ORs occurring in adverbial clauses. This discrepancy may account for why the data patterns found in our study appear incompatible with the corpus results reported in Wu et al.'s study, because structural parsing might tune to a finer or coarser-grained structure than the level of structure examined in Wu et al.'s study. In addition, further complications should be noted given differences in the definition of “animacy” adopted in Wu et al. and the current study. In Wu et al. (2009), classification of NPs was based on detailed semantic features. For example, an organization or institution NP (e.g., *Washington* or *Beijing*) was considered an animate NP when some degree of agency was associated with it, but the same type of NP was considered inanimate when it was used as a location^4^. In the current study, half of the stimuli (10 out of 20 items) involved organization or location NPs, but they were always considered inanimate NPs, regardless of whether their interpretations involved an agent reading (i.e., an agent of an action predicated within a relative clause) or a simple location reading. This may have affected the LA and HA ratings of these sentences. However, Wu et al. did not provide detailed information on how many of the NPs classified as animate were organizations or institutions. Given these limitations, it is not easy to clearly compare the results in the current study to the corpus results reported in Wu et al. Thus, further studies are needed for a proper evaluation of the Tuning Hypothesis in Chinese relative clause attachment.

Now, we turn to the question of why animacy matters in the structural analysis of sentences with relative clauses in Chinese. As previously discussed, the importance of animacy has been demonstrated in the processing of relative clauses. For example, Wu et al. (2012) showed that animacy of NPs affected the processing difficulty of SR and OR sentences in Chinese (cf. Hsiao and Gibson, 2003; Lin, 2006; Lin and Bever, 2006; Chen et al., 2008; Lin and Garnsey, 2010; Gibson and Wu, 2011; Vasishth et al., 2013). The current study also showed a clear animacy effect, but the resulting patterns were rather complicated. That is, Experiment 1 showed that relative clause attachment was modulated by animacy of a potential head NP such that an animate NP was more likely than an inanimate NP to be modified by a relative clause. Likewise, the results of Experiment 2 showed that this animate NP modification tendency interacted with relative clause type such that the animate NP modification tendency was limited to the SR condition. We accounted for these results in terms of the interaction between the animacy effect and other factors, such as the grammatical functions of a head NP within a relative clause and a default LA tendency (Shen, 2006). The subject grammatical role is typically associated with animate NPs, as an animate NP is more likely than an inanimate NP to be an agent or experiencer (Gennari and MacDonald, 2008). Thus, SRs are likely to involve an animate head NP, which leads to a stronger LA tendency (NP1 modification) in the SR Animate-Inanimate condition than in the SR Inanimate-Animate condition. On the other hand, the default strategy in Chinese seems to be an LA, as reported in Shen (2006) and supported by the results of Experiment 1 showing more LA responses overall despite animacy manipulations. Given this, the strong LA tendency in the SR condition could be due to a combined effect of the default LA tendency and the animate NP modification tendency in SRs. It is also possible that the SR Inanimate-Animate condition showed a weaker LA attachment bias because the animate NP modification tendency in SRs (i.e., NP2 attachment in this condition) conflicted with the default LA tendency (i.e., NP1 attachment).

The results of the OR conditions seem more complicated to account for. Given that head NPs in ORs in Chinese are more likely to be inanimate than animate (Pu, 2007; Wu et al., 2010), we initially predicted an HA tendency (NP2 modification) for the OR Animate-Inanimate condition and an LA tendency (NP1 modification) for the OR Inanimate-Animate condition. However, the OR conditions showed a weak LA tendency regardless of animacy ordering. Although this is consistent with the default LA tendency reported in Shen (2006), there was no clear evidence that relative clause attachment for ORs was modulated by animacy. It is important to note, however, that compared to a NP with an agent/experiencer role, a NP with a patient/theme role might be less semantically constrained for its animacy, as both animate and inanimate NPs tend to be good candidates for a patient/theme role (typically object roles). Indeed, more variations in the animacy of NPs have been noted in the object position than in the subject position (Dahl and Fraurud, 1996; Van Valin and LaPolla, 1997; Kempen and Harbusch, 2004; Bresnan et al., 2007; Dahl, 2008). Accordingly, this relative flexibility of animacy in the object position may have generally weakened the tendency to attach an OR to an inanimate head NP. It is not clear, however, why the apparently default LA effect was not clear in the ORs either. One possibility is that the institution/organization NPs used in the current study may not be typical head NPs and seldom occur in this structural position (cf. Desmet et al., 2006; Wu et al., 2010). Alternatively, the strong preference against animate NPs as head NPs of object relative clauses found in Pu (2007) and Wu et al. (2010) could in fact be a dispreference against agentivity rather than animacy (or humanness), as the object position is typically associated with theme or patient. Since both the animate NPs and inanimate institution/organization NPs used in this study can be associated with a certain level of agentivity, it is possible that neither of the NP types is favored as an attachment site for ORs, resulting in no clear animacy effect for ORs. If so, under this possibility, the significant animacy effect for SR suggests that the subject position is more sensitive to humanness than agentivity. While humanness and agentivity cannot be separated in most cases, the complicated semantic compositions of institution/organization NPs in this study might have affected relative clause attachment differently for SRs and ORs. Further studies are needed using various types of NPs to explore these possibilities and to confirm the general attachment tendency in relative clause sentences with an inanimate head NP in Chinese.

In summary, the present study investigated the effect of animacy in relative clause attachment in Chinese. Experiment 1 confirmed the previous finding of an LA preference in Chinese (Shen, 2006) with more LA responses than HA responses elicited overall in a production study. However, the results of Experiment 1 also showed a tendency to use a prenominal relative clause to modify an animate (rather than inanimate) NP in Chinese. In Experiment 2, using a comprehension task, we further showed that this animate NP modification tendency interacts with RC type, resulting in an animate NP modification tendency for SRs and no clear bias for ORs. These results are incompatible with purely structural parsing strategies such as Late Closure (Frazier, 1987) and the Predicate Proximity Principle (Gibson et al., 1996). Instead, like earlier studies that have shown sematic effects on relative clause attachment (Gilboy et al., 1995; Hemforth et al., 2000a,b, 2015), the current results suggest that attachment ambiguity resolution in Chinese relative clauses is sensitive to animacy as well as relative clause type.

## Ethics Statement

The studies involving human participants were reviewed and approved by the IRB committees of Konkuk University and Nanyang Technological University. The patients/participants provided their written informed consent to participate in this study.

## Author Contributions

NK conceived the study. DO and NK wrote the paper. DO, NK, and HC prepared experiments. DO and HC ran experiments. AZ prepared additional experiment and ran the experiment.

### Conflict of Interest Statement

The authors declare that the research was conducted in the absence of any commercial or financial relationships that could be construed as a potential conflict of interest.

## Footnotes

^1^Information on modification types is not available for the 20 participants recruited from Singapore, as hard copies of the questionnaires were lost during the relocation of the first author. Thus, the results presented in Table 3 are based on the responses from the 20 participants recruited from China. In particular, these results are based on 342 attachment responses. 78 unclassified responses were excluded.

^2^Descriptive relative clauses (e.g.,– 位非常勤奋的年轻教师 “a young teacher that works very hard”) are similar to subject relative clauses, as both types of relative clauses predicate the head noun. Descriptive relative clauses, however, contain stative intransitive verbs rather than verbs clearly taking arguments, as subject relative clauses do (Lin and Hu, in press). We propose the nature of the task (i.e., filling in a blank without response length requirements) is responsible for the predominance of short responses such as descriptive relative clauses.

^3^Summation of the ratings of LA and HA interpretations ranged from 4 to 14 (mean = 10.92, SD = 2.31).

^4^Personal communication with Fuyun Wu (Jan 2018).

## References

[B1] Baayen R. H. (2008). Analyzing Linguistic Data: A Practical Introduction to Statistics Using R. Cambridge: Cambridge University Press.

[B2] Baayen R. H. Davidson D. J. Bates D. M. (2008). Mixed-effects modeling with crossed random effects for subjects and items. J. Mem. Lang. 59, 390–412. 10.1016/j.jml.2007.12.005

[B3] Barr D. J. Levy R. Scheepers C. Tily H. J. (2013). Random effects structure for confirmatory hypothesis testing: keep it maximal. J. Mem. Lang. 68, 255–278. 10.1016/j.jml.2012.11.001 24403724 PMC3881361

[B4] Bates D. Mächler M. Bolker B. Walker S. (2015). Fitting linear mixed-effects models using lme4. J. Stat. Softw. 67, 1–48. 10.18637/jss.v067.i01

[B5] Bresnan J. Cueni A. Nikitina T. Baayen R. (2007). “Predicting the dative alternation,” in Proceedings of the LFG 01 Conference, eds Bouma G. Kramer I. Zwarts J. (Stanford, CA: CSLI Publications, 13–32.

[B6] Brysbaert M. Mitchell D. C. (1996). Modifier attachment in sentence processing: evidence from Dutch. Q. J. Exp. Psychol. 49A, 664–695. 10.1080/713755636

[B7] Carreiras M. Clifton C. (1993). Relative clause interpretation preferences in Spanish and English. Lang. Speech 36, 353–372. 10.1177/002383099303600401 8072346

[B8] Chen B. Ning A. Bi H. Dunlap S. (2008). Chinese subject-relative clauses are more difficult to process than the object-relative clauses. Acta Psychol. 129, 61–65. 10.1016/j.actpsy.2008.04.005 18538740

[B9] Corley M. M. B. (1995). The Role of Statistics in Human Sentence Processing. Doctoral dissertation, University of Exeter.

[B10] Cuetos F. Mitchell D. C. (1988). Cross-linguistic differences in parsing: restrictions on the use of the Late Closure strategy in Spanish. Cognition 30, 73–105. 10.1016/0010-0277(88)90004-2 3180704

[B11] Dahl O. (2008). Animacy and egophoricity: grammar, ontology and phylogeny. Lingua 118, 141–150. 10.1016/j.lingua.2007.02.008

[B12] Dahl O. Fraurud K. (1996). “Animacy in grammar and discourse,” in Reference and Referent Accessibility, eds Fretheim T. Gundel J. (Amsterdam: John Benjamins Publishing Company, 47–64.

[B13] Desmet T. Brysbaert M. De Baecke C. (2002). The correspondence between sentence production and corpus frequencies in modifier attachment. Q. J. Exp. Psychol. 55A, 879–896. 10.1080/02724980143000604 12188518

[B14] Desmet T. De Baecke C. Drieghe D. Brysbaert M. Vonk W. (2006). Relative clause attachment in Dutch: on-line comprehension corresponds to corpus frequencies when lexical variables are taken into account, Language and Cognitive Processes 21, 453–485. 10.1080/01690960400023485

[B15] Dussias P. (2001). “Sentence parsing in fluent Spanish-English bilinguals,” in One Mind, Two Languages: Bilingual Language Processing, ed Nicol J. L. (Cambridge, MA: Blackwell Publishers, 159–176.

[B16] Ehrlich K. Fernandez E. Fodor J. D. Stenshoel E. Vinereanu M. (1999). “Low attachment of relative clauses: new data from Swedish, Norwegian and Romanian,” in Poster Presented at the 12thAnnualCUNYConference on Human Sentence Processing (New York, NY).

[B17] Felser C. Robert L. Gross R. Marinis T. (2007). The processing of ambiguous sentences by first and second language learners of English. Appl. Psycholinguist. 24, 453–489. 10.1017/S0142716403000237

[B18] Fernández E. (2000). Bilingual Sentence Processing: Relative Clause Attachment in English and Spanish. Doctoral dissertation, CUNY Graduate Center.

[B19] Fodor J. D. (1998). Learning to parse. J. Psycholinguist. Res. 27, 285–319. 10.1023/A:1023258301588

[B20] Fodor J. D. (2002). “Prosodic disambiguation in silent reading,” in Proceedings of the North East Linguistic Society (NELS), 32, ed Hirotani M. (Amherst, MA: GLSA Publications, 112–132.

[B21] Frazier L. (1987). “Sentence processing: a tutorial review,” in Attention and Performance XII: The Psychology of Reading, ed Coltheart M. (Hillsdale, NJ: Erlbaum), 559–586.

[B22] Frenck-Mestre C. Pynte J. (2000). “Resolving syntactic ambiguities: cross-linguistic differences? in Cross-Linguistic Perspectives on Language Processing, eds De Vincenzi M. Lombardo V. (Dordrecht: Kluwer Academic Publishers, 119–148.

[B23] Gennari S. P. MacDonald M. C. (2008). Semantic indeterminacy in object relative clauses. J. Mem. Lang. 58, 161–187. 10.1016/j.jml.2007.07.004 19724662 PMC2735264

[B24] Gibson E. Pearlmutter N. J. Canseco-Gonzales E. Hickok G. (1996). Recency preference in the human sentence processing mechanism. Cognition 59, 23–59. 10.1016/0010-0277(95)00687-7 8857470

[B25] Gibson E. Wu H.-H. (2011). Processing Chinese relative clauses in context. Lang. Cogn. Process. 28, 125–155. 10.1080/01690965.2010.536656

[B26] Gilboy E. Sopena J. M. Clifton C. Frazier L. (1995). Argument structure and association preference in Spanish and English complex NPs. Cognition 54, 131–167. 10.1016/0010-0277(94)00636-Y 7874875

[B27] Grillo N. Costa J. (2014). A novel argument for the universality of parsing principles. Cognition 133, 156–187. 10.1016/j.cognition.2014.05.019 25032904

[B28] Gutiérrez-Ziardegi E. Carreiras M. Laka I. (2004). “Who was on the balcony? Bilingual Sentence processing: relative clause attachment in Basque and Spanish,” in CUNY Conference on Human Sentence Processing (College Park, MA: University of Maryland).

[B29] Hemforth B. Fernandez S. Clifton C. Frazier L. Konieczny L. Walter M. (2015). Relative clause attachment in German, English, Spanish and French: effects of position and length. Lingua 166A, 43–64. 10.1016/j.lingua.2015.08.010

[B30] Hemforth B. Konieczny L. Scheepers C. (2000a). “Syntactic attachment and anaphor resolution: the two sides of relative clause attachment,” in Architecture and Mechanisms of Language Processing, eds Crocker M. Pickering M. Clifton C. Jr. (Cambridge: Cambridge University Press, 259–281.

[B31] Hemforth B. Konieczny L. Scheepers C. (2000b). “Modifier attachment: relative clauses and coordinations,” in German Sentence Processing, eds Hemforth B. Konieczny L. (Dordrecht: Kluwer, 161–186.

[B32] Hsiao F. Gibson E. (2003). Processing relative clauses in Chinese. Cognition 90, 3–27. 10.1016/S0010-0277(03)00124-0 14597268

[B33] Jaeger T. F. (2008). Categorical data analysis: away from ANOVAs (transformation or not) and towards logit mixed models. J. Mem. Lang. 59, 434–446. 10.1016/j.jml.2007.11.007 19884961 PMC2613284

[B34] Jun S.-A. (2003). Prosodic phrasing and attachment preferences. J. Psycholinguist. Res. 32, 219–249. 10.1023/A:1022452408944 12690832

[B35] Jun S.-A. (2010). The implicit prosody hypothesis and overt prosody in English. Lang. Cogn. Process. 25, 1201–1233. 10.1080/01690965.2010.503658

[B36] Jun S. A. Kim S. (2004). “Default phrasing and attachment preferences in Korean,” in Proceedings of Interspeech-ICSLP (Jeju-si).

[B37] Kamide Y. Mitchell D. C. (1997). Relative clause attachment: nondeterminism in Japanese parsing. J. Psycholinguist. Res. 26, 247–254. 10.1023/A:1025017817290

[B38] Kempen G. Harbusch K. (2004). “A corpus study into word order variation in German subordinate clauses: animacy affects linearization independently of grammatical function assignment,” in Multidisciplinary Approaches to Language Production, eds Pechmann T. Habel C. (Berlin: Mouton de Gruyter),173–181.

[B39] Konieczny L. Hemforth B. (2000). “Modifier attachment in German: relative clauses and prepositional phrases,” in Reading as a Perceptual Process, eds Kennedy A. Radach R. Heller D. Pynte J. (Amsterdam: Elsevier, 517–528.

[B40] Kwon N. Kluender R. Kutas M. Polinsky M. (2013). Subject/object processing asymmetries in Korean relative clauses: evidence from ERP data. Language 89, 537–585. 10.1353/lan.2013.0044 25400303 PMC4231604

[B41] Kwon N. Lee Y. Gordon P. C. Kluender R. Polinsky M. (2010). Cognitive and linguistic factors affecting subject/object asymmetry: an eye-tracking study of pronominal relative clauses in Korean. Language 86, 546–582. 10.1353/lan.2010.0006

[B42] Lee D. Kweon S. (2004). A sentence processing study of relative clause in Korean with two attachment sites. Discourse Cogn. 11, 126–141.

[B43] Li C. N. Thompson S. A. (1989). Mandarin Chinese: A Functional Reference Grammar. Berkeley, CA: University of California Press.

[B44] Lin C.-J. (2006). Relative-Clause Processing in Typologically Distinct Languages: A Universal Parsing Account. Doctoral dissertation, University of Arizona, Tucson, AZ.

[B45] Lin C.-J. C Bever T. G. (2006). “Subject preference in the processing of relative clauses in Chinese,” in Proceedings of the 25th West Coast Conference on Formal Linguistics, eds Baumer D. Montero D. Scanlon M. (Somerville, MA: Cascadilla Proceedings Project), 254–260.

[B46] Lin C.-J. Hu H. (in press). “Linking comprehension production: Frequency distribution of Chinese relative clauses in the Sinica Treebank,” in Text, Speech, Language Technology Series, eds Huang C.-R. Hsieh S. Jin P. (Springer).

[B47] Lin Y. Garnsey S. M. (2010). “Animacy and the resolution of temporary ambiguity in relative clause comprehension in Mandarin,” in Processing and Producing Head-Final Structures, eds Yamashita H. Hirose Y. Packard J. (Dordrecht: Springer, 241–275.

[B48] Mak W. M. Vonk W. Schriefers H. (2002). The influence of animacy on relative clause processing. J. Mem. Lang. 47, 50–68. 10.1006/jmla.2001.2837

[B49] Mitchell D. C. Brysbaert M. (1998). “Challenges to recent theories of crosslinguistic variation in parsing: evidence from Dutch,” in Syntax and Semantics: A Crosslinguistic Perspective, ed Hillert D. (San Diego, CA: Academic Press, 313–335.

[B50] Mitchell D. C. Cuetos F. (1991). “The origins of parsing strategies,” in Current Issues in Natural Language Processing, ed Smith C. (Austin: University of Texas, Centre for Cognitive Science), 1–12.

[B51] Mitchell D. C. Cuetos F. Corley M. M. B. Brysbaert M. (1995). Exposure-based models of human parsing: evidence for the use of coarse-grained (nonlexical) statistical records. J. Psycholinguist. Res. 24, 469–488. 10.1007/BF02143162

[B52] Miyamoto E. T. (1998). Relative Clause Processing in Brazilian Portuguese and Japanese. Doctoral dissertation, Massachusetts Institute of Technology, Cambridge, 1998.

[B53] Miyamoto E. T. Nakamura M. Takahashi S. (2004). “Processing relative clauses in Japanese with two attachment sites,” in Proceedings of the North East Linguistic Society (NELS), 34, eds Moulton K. Wolf M. (Amherst, MA: GLSA Publications, 441–452.

[B54] Palmer M. Chiou F.-D. Xue N. Lee T.-K. (2005). Chinese Treebank 5.0. Philadelphia, PA: Linguistic Data Consortium.

[B55] Papadopoulou D. Clahsen H. (2003). Parsing strategies in L1 and L2 sentence processing: a study of relative clause attachment in Greek. Stud. Second Lang. Acquisit. 24, 501–528. 10.1017/S0272263103000214

[B56] Pu M. M. (2007). The distribution of relative clauses in Chinese discourse. Discourse Process. 43, 25–53. 10.1080/01638530709336892

[B57] Pynte J. (1998). “The time course of attachment decisions: evidence from French,” in Sentence Processing: A Crosslinguistic Perspective, eds Hillert D. (San Diego, CA: Academic Press, 227–245.

[B58] Quinn D. Abdelghany H. Fodor J. D. (2000). “More evidence of implicit prosody in silent reading: French, English and Arabic relative clauses,” in Poster Presented at 13th Annual CUNY Conference on Human Sentence Processing (San Diego, CA).

[B59] R Core Team (2018). R: A Language and Environment for Statistical Computing. R Foundation for Statistical Computing, Vienna Available onlne at: https://www.R-project.org/

[B60] Shen X. (2006). Late Assignment of Syntax Theory: Evidence From Chinese and English. Doctoral dissertation, University of Exeter.

[B61] Sturt P. Branigan H. Matsumoto-Sturt Y. (1999). “The effect of clausal and thematic domains on left branching attachment ambiguities,” in Proceedings of the 21st Annual Conference of the Cognitive Science Society (Mahwah, NJ), 718–723.

[B62] Van Valin R. LaPolla R. (1997). Syntax: Structure, Meaning and Function. Cambridge: Cambridge University Press.

[B63] Vasishth S. Agnihotri R. K. Fernández E. M. Bhatt R. (2004). “Noun modification preferences in Hindi,” in The Proceedings of Seminar on Construction of Knowledge (Udaipur: Vidya Bhawan Education Resource Centre), 160–171.

[B64] Vasishth S. Chen Z. Li Q. Guo G. (2013). Processing Chinese relative clauses: evidence for the subject-relative advantage. PLoS ONE 8:e77006. 10.1371/journal.pone.0077006 24098575 PMC3788747

[B65] Wu F. Kaiser E. Andersen E. (2009). “Animacy preference effects in Chinese relative clause processing,” in Proceedings of the Western Conference on Linguistics (WECOL), eds Grosvald M. Soares D. (Davis, CA: University of California), 320–239.

[B66] Wu F. Kaiser E. Andersen E. (2010). “Subject preference, head animacy, and lexical cues: a corpus study of relative clauses in Chinese,” in Processing and Producing Head-Final Structures, eds Yamashita H. Hirose Y. Packard J. (Dordrecht: Springer, 173–194.

[B67] Wu F. Kaiser E. Andersen E. (2012). Animacy effects in Chinese relative clause processing. Lang. Cogn. Process. 27, 1489–1524 10.1080/01690965.2011.614423

[B68] Yao B. Scheepers C. (2018). Direct speech quotations promote low relative-clause attachment in silent reading of English. Cognition 176, 248–254. 10.1016/j.cognition.2018.03.017 29609099

[B69] Zagar D. Pynte J. Rativeau S. (1997). Evidence for early-closure attachment on first-pass reading times in French. Q. J. Exp. Psychol. 50A, 421–438. 10.1080/713755715

