# Supplementary material for: The Role of Animacy and Structural Information in Relative Clause Attachment: Evidence From Chinese
Source: Front Psychol. 2019 Jul 17;10:1576. doi: 10.3389/fpsyg.2019.01576 (PMC6650783; doi:10.3389/fpsyg.2019.01576)
Supplement: Supplementary file 1 [file Presentation_1.pdf]

Appendix

Stimuli sentences used in Experiment 2

1 SR ani-ina 多亏了宣扬爱国主义的导演的电影，电视台成功地化解了这场危机。
1 SR ina-ani 多亏了宣扬爱国主义的电影的导演，电视台成功地化解了这场危机。
1 OR ani-ina 多亏了观众认可的导演的电影，电视台成功地化解了这场危机。
1 OR ina-ani 多亏了观众认可的电影的导演，电视台成功地化解了这场危机。
2 SR ani-ina 在泄漏了机密的记者的报社旁，有许多可疑人物出现。
2 SR ina-ani 在泄漏了机密的报社的记者旁，有许多可疑人物出现。
2 OR ani-ina 在我讨厌的记者的报社旁，有许多人在抗议。
2 OR ina-ani 在我讨厌的报社的记者旁，有许多人在抗议。
3 SR ani-ina 关于批判社会不良风气的诗人的诗歌，读者间起了一些争议。
3 SR ina-ani 关于批判社会不良风气的诗歌的诗人，读者间起了一些争议。
3 OR ani-ina 关于很多人喜欢的诗人的诗歌，读者间起了一些争议。
3 OR ina-ani 关于很多人喜欢的诗歌的诗人，读者间起了一些争议。
4 SR ani-ina 除了欺负这些小公司的老板的企业，我们还有好一些竞争对手。
4 SR ina-ani 除了欺负这些小公司的企业的老板，我们还有好一些竞争对手。
4 OR ani-ina 除了我们邀请来参加年会的老板的企业，没有其他商业伙伴前来拜访。
4 OR ina-ani 除了我们邀请来参加年会的企业的老板，没有其他商业伙伴前来拜访。
5 SR ani-ina 在获得政府补贴的农夫的农场旁，有一块巨大的德语广告牌。
5 SR ina-ani 在获得政府补贴的农场的农夫旁，有一块巨大的德语广告牌。
5 OR ani-ina 在我喜欢的农夫的农场旁，有一块巨大的德语广告牌。
5 OR ina-ani 在我喜欢的农场的农夫旁，有一块巨大的德语广告牌。
6 SR ani-ina 在备受青年女性欢迎的司机的汽车周围，有一些路人围了过来。
6 SR ina-ani 在备受青年女性欢迎的汽车的司机周围，有一些路人围了过来。
6 OR ani-ina 在警察拦住的司机的汽车周围，有一些路人围了过来。
6 OR ina-ani 在警察拦住的汽车的司机周围，有一些路人围了过来。
7 SR ani-ina 通过捧红这些名模的摄影师的杂志，读者们能接触到最新时尚。
7 SR ina-ani 通过捧红这些名模的杂志的摄影师，读者们能接触到最新时尚。
7 OR ani-ina 通过女明星自己选择的摄影师的杂志，读者们能看到她不同的一面。
7 OR ina-ani 通过女明星自己选择的杂志的摄影师，读者们能看到她不同的一面。
8 SR ani-ina 至于失去客户的经理的公关部门，公司自有安排。
8 SR ina-ani 至于失去客户的公关部门的经理，公司自有安排。
8 OR ani-ina 至于经理看重的员工的部门，公司自有安排。
8 OR ina-ani 至于经理看重的部门的员工，公司自有安排。
9 SR ani-ina 对于获得大奖的那个画家的山水画，大家产生了极大的兴趣。
9 SR ina-ani 对于获得大奖的那个山水画的画家，大家产生了极大的兴趣。
9 OR ani-ina 对于媒体宣传的那个画家的山水画，大家产生了极大的兴趣。
9 OR ina-ani 对于媒体宣传的那个山水画的画家，大家产生了极大的兴趣。

10 SR ani-ina 对于获得媒体关注的那个医生的医院，我知之甚少。
10 SR ina-ani 对于获得媒体关注的那个医院的医生，我知之甚少。
10 OR ani-ina 对于病人赞扬的那个医生的医院，我知之甚少。
10 OR ina-ani 对于病人赞扬的那个医院的医生，我知之甚少。
11 SR ani-ina 对于获奖的那个编剧的剧本，我们了如指掌。
11 SR ina-ani 对于获奖的那个剧本的编剧，我们了如指掌。
11 OR ani-ina 对于导演挑选的那个编剧的剧本，我们了如指掌。
11 OR ina-ani 对于导演挑选的那个剧本的编剧，我们了如指掌。
12 SR ani-ina 关于享有盛名的那个雕刻家的雕塑，我一直很感兴趣。
12 SR ina-ani 关于享有盛名的那个雕塑的雕刻家，我一直很感兴趣。
12 OR ani-ina 关于法国人很欣赏的那个雕刻家的雕塑，我也一直很感兴趣。
12 OR ina-ani 关于法国人很欣赏的那个雕塑的雕刻家，我也一直很感兴趣。
13 SR ani-ina 依据备受关注的老师的学校的回应，警方将处理这次校园暴力事件。
13 SR ina-ani 依据备受关注的学校的老师的回应，警方将处理这次校园暴力事件。
13 OR ani-ina 依据记者报道的老师的学校的回应，警方将处理这次校园暴力事件。
13 OR ina-ani 依据记者报道的学校的老师的回应，警方将处理这次校园暴力事件。
14 SR ani-ina 在赢得顾客点赞的老板的餐馆那里，我看到了一幅价值连城的画。
14 SR ina-ani 在赢得顾客点赞的餐馆的老板那里，我看到了一幅价值连城的画。
14 OR ani-ina 在马云赞助的老板的餐馆那里，我看到了一幅价值连城的画。
14 OR ina-ani 在马云赞助的餐馆的老板那里，我看到了一幅价值连城的画。
15 SR ani-ina 多亏了捐赠这些书籍的牧师的教会，这个社区修建了一个迷你图书馆。
15 SR ina-ani 多亏了捐赠这些书籍的教会的牧师，这个社区修建了一个迷你图书馆。
15 OR ani-ina 多亏了这些志愿者帮助的牧师的教会，当地儿童才得到足够的资金与支持。
15 OR ina-ani 多亏了这些志愿者帮助的教会的牧师，当地儿童才得到足够的资金与支持。
16 SR ani-ina 从赢得游客好评的房东的房子那里，我得知一个天大的秘密。
16 SR ina-ani 从赢得游客好评的房子的房东那里，我得知一个天大的秘密。
16 OR ani-ina 从这些游客赞美的房东的房子那里，我得知一个天大的秘密。
16 OR ina-ani 从这些游客赞美的房子的房东那里，我得知一个天大的秘密
17 SR ani-ina 通过揭露惊天秘密的摄影师的照片，大家进一步了解了日军侵华历史。
17 SR ina-ani 通过揭露惊天秘密的照片的摄影师，大家进一步了解了日军侵华历史。
17 OR ani-ina 通过日本人痛恨的摄影师的照片，大家进一步了解了日军侵华历史。
17 OR ina-ani 通过日本人痛恨的照片的摄影师，大家进一步了解了日军侵华历史。
18 SR ani-ina 对荣获大奖的演员的歌舞剧，观众们个个都赞赏不已。
18 SR ina-ani 对荣获大奖的歌舞剧的演员，观众们个个都赞赏不已。
18 OR ani-ina 因为大家都喜欢的这名演员的歌舞剧，许多学生参加了歌舞团体。
18 OR ina-ani 因为大家都喜欢的这部歌舞剧的演员，许多学生参加了歌舞团体。
19 SR ani-ina 多亏了阐述共产主义的作者的文章，大家对革命事业充满了希望。
19 SR ina-ani 多亏了阐述共产主义的文章的作者，大家对革命事业充满了希望。
19 OR ani-ina 多亏了教授推荐的作者的文章，大家对接下来的实验充满了信心。
19 OR ina-ani 多亏了教授推荐的文章的作者，大家对接下来的实验充满了信心。

20 SR ani-ina 多亏了讲述明朝历史的这个作家的这部小说，学生们对明朝历史产生了浓厚的兴趣。
20 SR ina-ani 多亏了讲述明朝历史的这部小说的这个作家，学生们对明朝历史产生了浓厚的兴趣。
20 OR ani-ina 多亏了历史老师推荐的这个作家的这部小说，学生们对明朝历史产生了浓厚的兴趣。
20 OR ina-ani 多亏了历史老师推荐的这部小说的这个作家，学生们对明朝历史产生了浓厚的兴趣。
